# *Modicellaguangxiensis* (Mortierellomycota, Mortierellaceae), a new species from south-western karst areas of China

**DOI:** 10.3897/BDJ.12.e115044

**Published:** 2024-01-19

**Authors:** Guang-Fu Mou, Tolgor Bau

**Affiliations:** 1 Jilin Agricultural University, Changchun, China Jilin Agricultural University Changchun China; 2 Guangxi Key Laboratory of Plant Conservation and Restoration Ecology in Karst Terrai, Guangxi Institute of Botany, Guangxi Zhuang Autonomous Region and Chinese Academy of Sciences, Guilin, China Guangxi Key Laboratory of Plant Conservation and Restoration Ecology in Karst Terrai, Guangxi Institute of Botany, Guangxi Zhuang Autonomous Region and Chinese Academy of Sciences Guilin China; 3 Nonggang Karst Ecosystem Observation and Research Station of Guangxi, Chongzuo, China Nonggang Karst Ecosystem Observation and Research Station of Guangxi Chongzuo China

**Keywords:** new taxon, taxonomy, phylogeny, sporocarp-forming fungi

## Abstract

**Background:**

*Modicella* Kanouse (1936) is the only genus of Mortierellaceae known to produce macroscopic fruiting bodies in the form of small, whitish, round sporocarps. Specimens which belong to *Modicella* were collected during our field investigations in tropical karst areas of China. Based on morphological characteristics and phylogenetic analysis, a new species is introduced.

**New information:**

*Modicellaguangxiensis* is described as a new species from tropical karst areas of China. The main distinguishing characteristics of *M.guangxiensis* are the number of sporangiospores per sporangium (11–18), the size of sporangiospores (14–34 × 12–27.5 μm) and the surface of some hyphae with hemispherical tuber. The phylogenetic analyses, based on the internal transcribed spacer (ITS) and the large subunit (LSU) regions of rDNA sequences using Bayesian (BA) and Maximum Likelihood (ML) methods showed that the new taxon is closely related to *M.reniformis*.

## Introduction

*Modicella* Kanouse is a small genus of Mortierellaceae, typified by the species *Modicellamalleola* (Harkn.) Gerd. & Trappe. This genus is the only genus of Mortierellaceae known to produce macroscopic fruiting bodies in the form of small, whitish, round sporocarps. They are morphologically Mortierellaceae-like in their acolumellate sporangium and garlic-like odour that is similar to that of other Mortierellaceae species (Vandepol et al. 2020).

At present, only three taxa have been recognised in *Modicella* (http://www.indexfungorum.org), namely *M.albostipitata* J.A. Cooper, *M.malleola* (Harkn.) Gerd. & Trappe and *M.reniformis* (Bres.) Gerd. & Trappe. The species of *Modicella* have usually been found on soil or within decomposing plant materials. *M.albostipitata* was found in New Zealand (Cooper and Park 2020); *M.malleola* has been collected in Europe, North America and China; *M.reniformis* has been collected in Brazil, Chile and Argentina (Smith et al. 2013).

During our field investigations in tropical karst areas of China, a sporocarp-forming fungus was discovered. Morphological study and phylogenetic analysis, based on ITS and LSU rDNA sequences, proved that it is new to science.

## Materials and methods

### Sampling, morphological observations and descriptions

One specimen was collected from the Guangxi Zhuang Autonomous Region, China and was dried in silica gel. Dried specimen preserved in the Herbarium of Guangxi Institute of Botany (IBK). Macroscopic characteristics are based on fresh specimens. Microscopic characteristics were obtained, based on dried specimens and examined with a light microscope (Olympus BX43F, Japan). Colour microscopic photos were taken by Mshot camera (Mshot MDX6-T, China). Measurements were made on the tissues mounted in purified water. Tissues were stained with 1% Congo Red solution or Lactate Carbolic Cotton Blue. The size of sporangia and sporangiospores was calculated, based on measurements of randomly sampled 42 sporangia and 44 sporangiospores.

### DNA extraction, PCR amplification and sequencing

Total DNA was extracted from dried specimens using a NuClean Plant Genomic DNA kit (CWBIO). ITS4/ITS5 and LR0R/LR7 were taken as primer sequences to amplify the ITS and LSU regions (White et al. 1990). The PCR procedure for ITS and LSU was as follows: initial denaturation at 94℃ for 4 min, followed by 35 cycles at 94℃ for 40 s, 52℃ for 40 s, 72℃ for 1 min and final extension of 72℃ for 10 min. The DNA sequencing was completed by Shenggo Biological Technology Co. Ltd. Sequences derived in this study were deposited in GenBank (http://www.ncbi.nlm.nih.gov/genbank).

### Data analysis

A total of 11 *Modicella* and allied species’ sequences of ITS and LSU (including the new taxon) were used for molecular phylogenetic analyses, while sequences retrieved from GenBank (Table 1) mainly refer to Smith et al. (2013), Wagner et al. (2013) and Cooper and Park (2020). *Actinomortierellacapitata* (CBS 859.70), *A.capitata* (CBS 110640), *A.wolfii* (CBS 209.69) and *A.wolfii* (CBS 612.70) were used as the outgroup, based on the earlier studies of Vandepol et al. (2020) and a recent bioRxiv preprint studies of Zhao et al. (2022).

Sequences of ITS and LSU were aligned separately with online MAFFT (Katoh et al. 2017) using the default settings. Prior to phylogenetic analysis, ambiguous sequences at the start and the end were deleted and gaps were manually adjusted to optimise the alignment using the default parameters in BioEdit v.7.2.5. (Hall 1999). The ITS and LSU sequences were concatenated as a combined file using SequenceMatrix (Vaidya et al. 2011). MrModelTest v.2.3 was used to estimate the optimal model (Nylander 2004). Bayesian Inference (BI) analysis was performed with MrBayes v.3.2.6 and four Markov Chains (MCMC) were run for one million generations, sampling every 100^th^ generation (Ronquist and Huelsenbeck 2003). Maximum Likelihood (ML) bootstrap analysis was performed with rapid bootstrapping algorithm and 1000 replicates, followed by a ML tree search in raxmlGUI 2.0 (Edler et al. 2020). The tree was visualised by FigTree v.1.4.3 (Rambaut 2012). The final concatenated sequence alignments were deposited in TreeBase (https://www.treebase.org/treebase-web/home.html) with the submission ID 30895.

## Taxon treatments

### Modicella guangxiensis

T. Bau & G. F. Mou sp. nov.

F875AD3D-49AD-5F72-877A-9530C15231BE

FN 571583

#### Materials

- **Type status:** Holotype. **Occurrence:** occurrenceID: 4499BC04-583D-535F-A55A-331F2F84111A; **Taxon:** kingdom: Fungi; phylum: Mortierellomycota; class: Mortierellomycetes; order: Mortierellales; family: Mortierellaceae; genus: Modicella; taxonRank: species; **Location:** country: China; stateProvince: Guangxi; county: Longzhou; verbatimElevation: 191 m; verbatimLatitude: 22^◦^27′53.59″ N; verbatimLongitude: 106^◦^55′38.42″ E; **Identification:** identifiedBy: Guang-Fu Mou; **Record Level:** type: Nonggang National Nature Reserve, on soil or decomposing plant materials, 15 August 2021, Guang-fu Mou, M2021081545 (holotype IBK!).; institutionID: IBK; collectionID: M2021081545; institutionCode: Herbarium of Guangxi Institute of Botany (IBK)

#### Description

**Figure 1.**
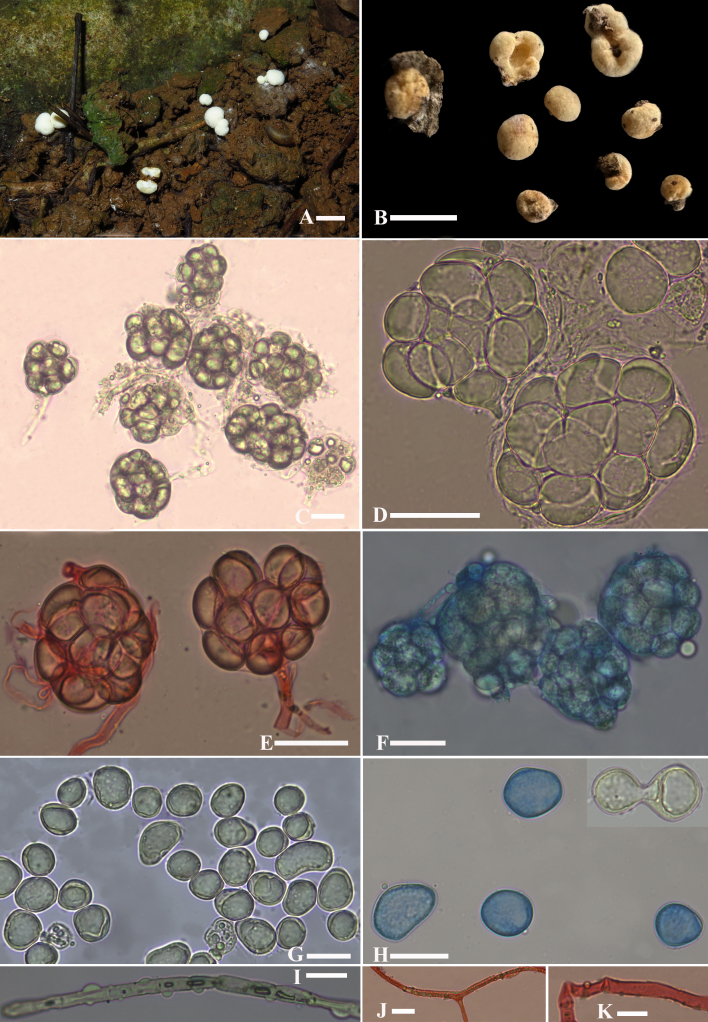
Morphology of *Modicellaguangxiensis*. **A** Sporocarps and its habitat; **B** Dried yellowish sporocarps; **C–F** Intact sporangia; **G, H** Sporangiospores; **I, J, K** Different kinds of hyphae. Scale bar A–B = 5 mm; C–F = 25 μm; G–H = 20 μm; I–K = 10 μm. C, D, G, I. in purified water; E, J, K. in 1% Congo Red; F, H. in Cotton Blue; Photos by Guang-fu Mou, from M2021081545 (holotype IBK!).

Sporocarps hemispherical to nearly spherical, base flattened or flat with a cavity; 1.5–4.5 mm in diameter, 1.5–3.0 mm in height; white when fresh and becoming pale yellow when dried; fragile; surface appearing granular when fresh and containing sporangia (Fig. 1A and B). Peridium absent. Columella absent. Basal hyphae white. Odour not recorded.

Sporangia subglobose, ellipsoid, (41)45–62(68) × (35)40–54(61) μm, hyaline, thin-walled, containing 11–15(18) sporangiospores (Fig. 1C–F); each sporangium is attached to a single subtending hypha (Fig. 1E). Sporangiospores subglobose, ellipsoid, ovoid or irregular, colourless, cyanophilous, with a significant oil-like content, (14)16–30(34) × (12)14–23.5(27.5) μm, 21 × 17 μm on average (Fig. 1G and H); some sporangiospores will elongate and constrict into two sporangiospores (Fig. 1H). There are three kinds of hyphae: hyphae of surface with hemispherical tuber (Fig. 1 I), 3 μm in diameter, not easily found; hyphae of branched (Fig. 1J), 3–4.5 μm in diameter; hyphae linking with sporangium (Fig. 1K), wider in width, 5.5–6.8 μm, sometimes branched. All hyphae hyaline, thin-walled or slightly thick-walled, septa rarely seen. Zygospores not observed.

#### Diagnosis

Differing from the known species by the number of sporangiospores per sporangia (11–18), the size of sporangiospores (14–34 × 12–27.5 μm) and the surface of some hyphae with hemispherical tuber.

#### Etymology

*guangxiensis* (Lat.): referring to the locality of the type specimen, Guangxi Zhuang Autonomous Region of China.

#### Distribution

So far, only known from Guangxi (CHINA).

#### Ecology

It grows on broad-leaved forest soil or decomposing plant materials of karst areas.

## Analysis

### Phylogenetic analyses

**Figure 2.**
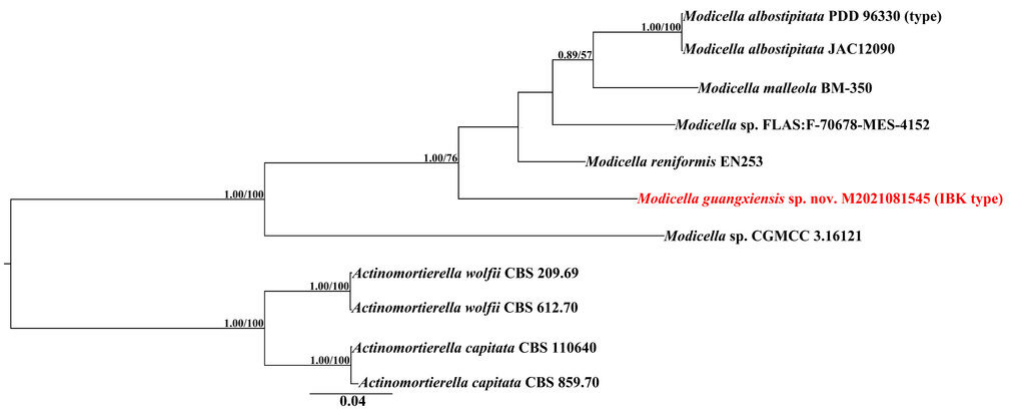
Phylogenetic tree inferred from partial ITS+LSU sequences showing phylogenetic relationships of *Modicellaguangxiensis*. Bayesian Inference (BPP ≥ 0.85) and Maximum Likelihood support values (BS ≥ 50) are shown (BPP/BS). New species is in red font.

The phylogenetic analyses were inferred from ITS+LSU ribosomal DNA datasets, which had 1635 (including gaps) base pairs. The ITS dataset had an alignment length of 694 (including gaps) base pairs representing 11 sequences. The LSU dataset had an alignment length of 941 (including gaps) base pairs representing eight sequences. The best-fit model used for Bayesian Inference (BI) analysis for the combined two-marker data subset (the two-marker dataset was treated individually) is the same, being the GTR+G model. For the Maximum Likelihood (ML) bootstrap analysis, the ITS and LSU datasets were treated as a whole, using the GTR+G model. The Bayesian Inference (BI) analysis and Maximum Likelihood (ML) bootstrap analysis obtained the same topology, this present manuscript showing the topology of Bayesian Inference (BI) analysis (Fig. 2). Maximum Likelihood bootstrap values (MLBS ≥ 50%) and posterior probabilities values for BI (BPP ≥ 0.85) are given above each branch (BPP/BS), with the new taxon being in red font.

In the ITS+LSU analyses (Fig. 2), our specimen (red font) is grouped together with the species of *Modicella* with full support values (1.00 BPP and 100% BS). Within the *Modicella* clade, our specimen (red font) is in a independent clade differing from all known species of *Modicella*, with full support values of BI analyses (1.00 BPP) and strong statistical support values of ML analyses (76% BS).

## Discussion

*Modicella* is a small genus and also the only genus that produces fruiting bodies in Mortierellaceae. Phylogenetically, our specimen separated from the known species and formed a single clade receiving strong statistical support (1.00/76) (Fig. 2), confirming it as a distinct taxon.

Morphologically, *M.guangxiensis* differs from *M.albostipitata* by the sporocarps being smaller and without a distinct pseudostipe, the fewer number of sporangiospores per sporangia (11–18 vs. > 100), the larger sporangiospores (14–34 × 12–27.5 μm vs. 10–16 μm) (Cooper and Park 2020). The new species differs from *M.malleola* by the larger sporangiospores [14–34 × 12–27.5 μm vs. 6.5–23 × 4–22 μm (Smith et al. 2013) or average 21 × 17 μm vs. 8–12 μm (Thaxter 1922)]; *M.guangxiensis* differs from *M.reniformis* by the greater number of sporangiospores per sporangia (11–18 vs. 3–8) (Smith et al. 2013). In addition, the surface of hyphae with hemispherical tuber also differs from the known species.

## Supplementary Material

XML Treatment for Modicella guangxiensis

## Figures and Tables

**Table 1.** Specimens used in phylogenetic analysis and GenBank accession numbers (the newly-generated sequences are in bold).

**Species**	**Voucher/Strain number**	**GenBank Accession numbers**	
		ITS	LSU
*Actinomortierellacapitata*	CBS 859.70	MH859983	MH871779
*Actinomortierellacapitata*	CBS 110640	JX975923	JX976163
*Actinomortierellawolfii*	CBS 209.69	MW577262	—
*Actinomortierellawolfii*	CBS 612.70	MH859876	MH871661
*Modicellaalbostipitata*	PDD 96330	NR_171266	NG_074467
*Modicellaalbostipitata*	JAC12090	MT649493	MT649494
***Modicellaguangxiensis***	**M2021081545**	**OR711265**	**OR710909**
*Modicellamalleola*	BM-350	KF053135	KF053131
*Modicellareniformis*	EN253	KF053136	KF053132
*Modicella* sp.	FLAS:F-70678-MES-4152	OP339685	—
*Modicella* sp.	CGMCC 3.16121	OL678160	—
